# Willingness to Adopt Telemedicine in Major Iraqi Hospitals: A Pilot Study

**DOI:** 10.1155/2015/136591

**Published:** 2015-10-08

**Authors:** Mohd Khanapi Abd Ghani, Mustafa Musa Jaber

**Affiliations:** Biomedical Computing and Engineering Technologies (BIOCORE) Applied Research Group, Faculty of Information and Communication Technology, Universiti Teknikal Malaysia Melaka, Durian Tunggal, 75450 Ayer Keroh, Malacca, Malaysia

## Abstract

The Iraqi healthcare services are struggling to regain their lost momentum. Many physicians and nurses left Iraq because of the current situation in the country. Despite plans of calling back the skilled health workforce, they are still worried by the disadvantages of their return. Hence, technology plays a central role in taking advantage of their profession through the use of telemedicine. Studying the factors that affect the implementation of telemedicine is necessary. Telemedicine covers network services, policy makers, and patient understanding. A framework that includes the influencing factors in adopting telemedicine in Iraq was developed in this study. A questionnaire was distributed among physicians in Baghdad Medical City to examine the hypothesis on each factor. The Statistical Package for the Social Sciences was utilized to verify the reliability of the questionnaire and Cronbach's alpha test shows that the factors have values more than 0.7, which are standard.

## 1. Introduction

Developing countries are using telemedicine to link health centers, tertiary centers, and referral hospitals. Several e-health applications, such as telemedicine, can be found with different degrees of success in majority of developing countries [1, 2]. According to [3], the following two factors explain the wide use of telemedicine: the lack of alternatives such as building hospitals in each village which is costly and (2) the apparent geographical superiority of telemedicine over traditional medicine. Despite high expectations from the technology, configuring its use into a certain routine is impossible [4].

The framework of adopting telemedicine in Iraq was categorized into four characteristics, namely, technological, organizational, environmental, and individual. Fourteen influencing factors were explicated, and each was included in its appropriate characteristic. This framework can enhance the quality of the healthcare sector in Iraq. Thus, the influencing factors are enablers in the decision-making and e-participation of hospitals [5]. This study illustrates a suitable design of the questionnaire on willingness to adopt telemedicine of telemedicine in Iraq to evaluate the framework. A pilot study was conducted to improve the reliability of the questionnaire.

Several benefits can be expected from the use of telemedicine. These benefits can affect not only the health providers and the patients, but also the entire healthcare organization. For example, a study on physicians and health managers in the Quebec health region in Canada by [6] found that the benefits and usefulness of the telemedicine system were seen on three levels: (1) clinical/patient, (2) professional/educational, and (3) organizational.

## 2. Limitations of the Research

This study aims to examine the feasibility and acceptance level telemedicine framework in Iraq. However, a number of limitations should be considered. First, studies regarding telemedicine in Iraq are limited, with few studies conducted by the Ministry of Health of Iraq. This situation caused some difficulties in designing the questionnaire. Second, the physicians' level of understanding of telemedicine is limited because telemedicine is a new technology in Iraq [5, 7]. Third, only a handful of physicians practice electronic consultation (e-consultation). Fourth, physicians have limited grasp and knowledge of data warehouse because it is viewed and considered as a new tool in telemedicine. Fifth, the number of respondents was 35 only. Finally, the unstable political climate and strife in Iraq added challenges to the scope and limitations of this study.

## 3. The Framework

The technology-organization-environment (TOE) framework, which was developed by [8], is chosen and applied as the framework of this study. [1, 2], These studies have demonstrated consistent support for TOE's ability to provide a comprehensive perspective on innovation adoption, while facilitating the flexibility to identify and categorize unique factors that may emerge in particular situations [9]. Moreover, the main reason for selecting this framework is that this approach has the potential to address issues of this study. On the subject of appropriate framework selection, [10] advocates a framework that has been adapted and adopted and requires further development and fine-tuning in its application for contextual matching.

This study adapted the TOE framework to improve its suitability in the field of telemedicine. This adaption considers technological, organizational, environmental, and individual characteristics. In addition, social exchange theory, diffusion of innovation theory, TOE adoption framework, and previous studies are employed to investigate the factors that influence telemedicine in Iraq. These theories have been applied in electronic information sharing studies [11, 12]. Social exchange theory refers to shared information in the public sector [13]. This theory is based on power and trust. Thus, factors such as top management support of upper-level leadership are employed. The focus of this theory is to help organizations and individuals in deciding whether to adopt or reject a new innovation and to estimate the duration of acceptance and use of a new technology. In reality, the theory has been used to clarify and evaluate a wide range of IT adoption, such as that of the Internet [14], database machine [15], software engineering techniques [16], and IT in general [17].

**Figure 1:**
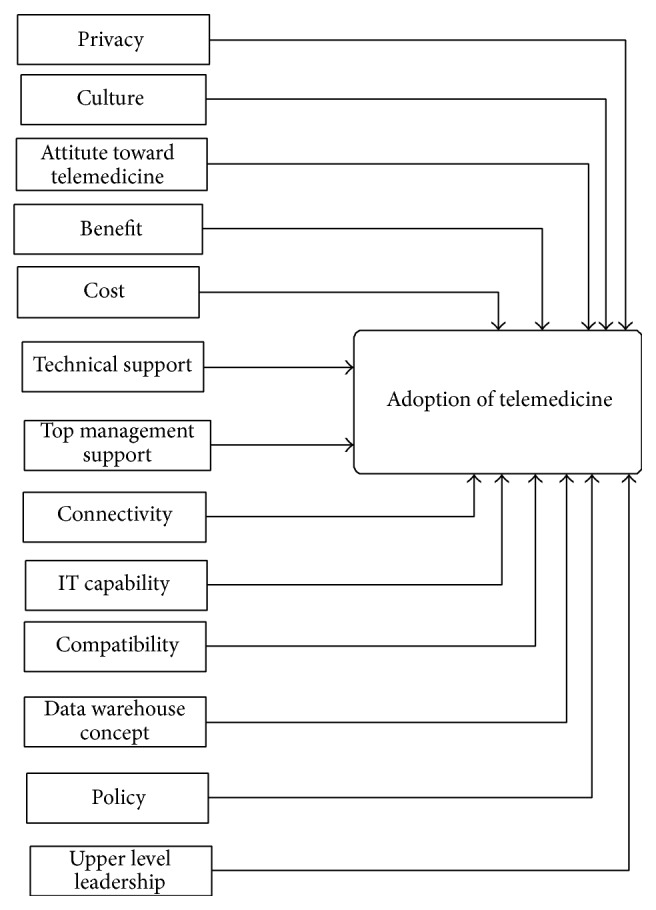
Framework of willingness to adopt telemedicine in Iraq.

The remaining factors have been adopted from researches done in Middle Eastern countries such as Kuwait and Jordan. Each characteristic has numerous influencing factors. The influencing factors of the current research are privacy, culture, attitude to telemedicine, benefit, connectivity, IT capability, compatibility, data warehouse concept, technical support, top management support, cost, approach, and upper-level leadership. This study also introduces 13 hypotheses. One hypothesis is formulated for every factor.  Figure 1 shows the framework of this study.

The factors that can promote engagement in the willingness to accept telemedicine have been identified. The pilot study is performed to evaluate the questionnaire items and to determine the appropriate questions for the survey. The questionnaires were distributed among physicians. The succeeding section explains the questionnaire design.

## 4. Research Methods

The survey method is employed in this study because surveys are prevalently used to begin reports and it is a suitable method for examining factors and hypotheses. The questionnaire methodology is used for data collection. Paper [18] shows that the questionnaire design relies on three criteria, namely, the manner by which the questions are written, planning for the classification of variables, and the appearance of the questionnaire. This study uses a six-part questionnaire. Part 1 includes the questions related to demographic factors. Part 2 relates to the state of telemedicine. Part 3 relates to the characteristics of telemedicine. Part 4 relates to organizational characteristics. Part 5 includes questions related to technological characteristics. Part 6 relates to environmental characteristics. Please refer to the Appendix.

The questionnaire is designed based on the content of each factor. Suggestions and advice from colleagues and supervisors are considered in improving the design of the questionnaire, as well as in developing the questionnaire. The questionnaire is written in English and then translated into Arabic, which is the official language in Iraq.  Table 1 shows the operationalization of the factors and items.

## 5. Pilot Study

Questionnaires were distributed among physicians who are working in Baghdad Medical City. Among the 35 questionnaires collected, only 5 were not correctly answered.  Table 2 illustrates the demographic characteristics of the pilot study.

### 5.1. Usage of Devices

**Figure 2:**
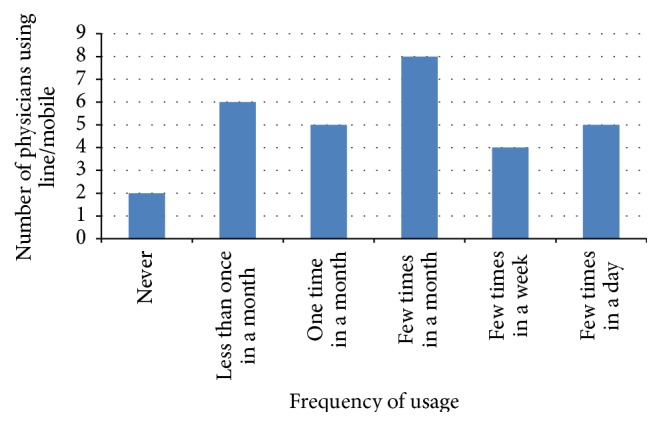
Line/mobile usage.

**Figure 3:**
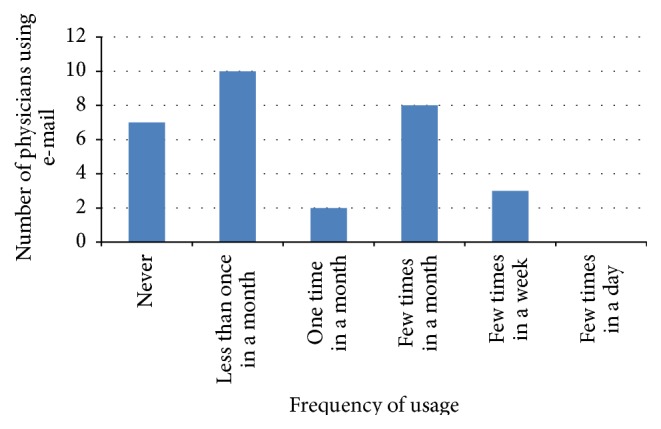
E-mail usage.

**Figure 4:**
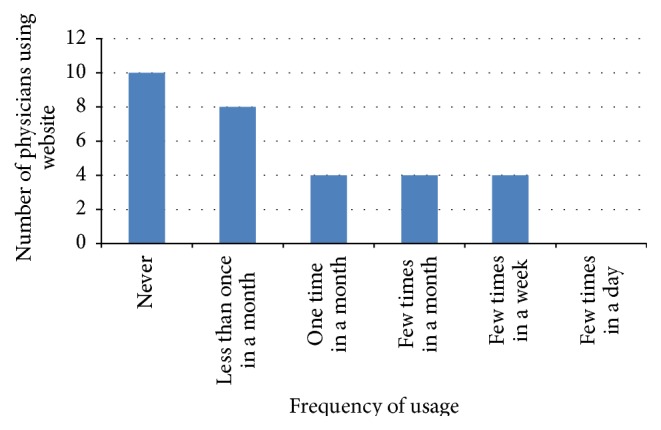
Website usage.

**Figure 5:**
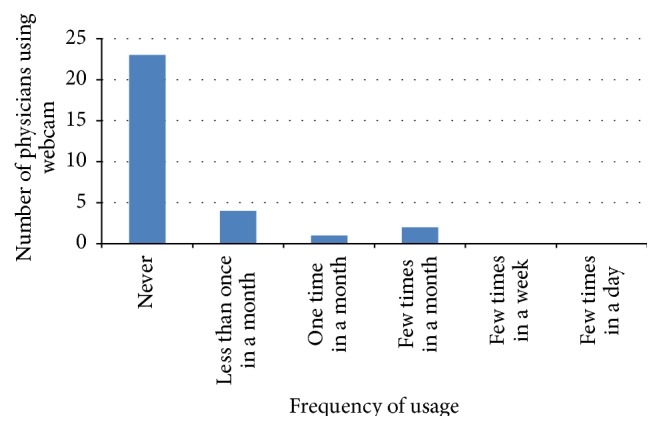
Webcam usage.

Queries on the method of how physicians conduct electronic consultation in inputting patient information were conducted in this study. Five methods were mentioned in the questionnaire, but the participants were provided an option to add another method that they have used. However, no other method of information sharing was added by the participants. Thus, each study reveals different percentages related to the five methods used. Figures 2, 3, 4, and 5 illustrate the percentages of the most used method of electronic consultation. The data collected reveal that e-mail is the most used method in electronic consultation.

The database was not utilized by the participants to access the ministry's information. The results from the two pilot studies reveal that the most used means of electronic consultation between physicians and patients are phone, e-mail, and website. The findings also show that the use of webcam is limited. The use of database was mentioned only once in both studies.

### 5.2. Percentage and Initiation Time of Sharing Information

The electronic information sharing practice in the Iraqi healthcare sector was measured in terms of the percentage of electronic consultation used and the time information consultation was initiated. Thus, based on the findings, the participants of the pilot studies illustrated the percentage of electronic consultation used. Most of the participants revealed that the percentage of consultation is 63.3% from 1% to 20% of their work. Most of the participants mentioned that electronic consultation was initiated between 1 year and 3 years ago.  Table 3 shows the consultation percentage per year was initiated.

## 6. Results of Data Analysis

The most commonly used test in reliability measurement of any pilot study questionnaire is Cronbach's alpha [18, 36]. According to [37], Cronbach's alpha test possesses values within the range of 0 to 1; a higher level of range indicates greater value of reliability. Values of 0.9 and above are excellent, 0.8 and above are good, 0.7 and above are acceptable, 0.6 and above are questionable, and less than 0.6 are poor [37, 38].

The data collected from the two pilot studies have been analyzed using Statistical Package for the Social Sciences 20 to identify the values of each factor in Cronbach's alpha. Based on the pilot study, no change should be made in the items of the pilot study. These items were rewritten to create a clear and easy-to-understand questionnaire. The items of the factors were also reduced. Thus, all factors have values of more than 0.7, which are acceptable.  Table 4 shows Cronbach's alpha and number of items for each factor.

## 7. Conclusion and Future Work

This study was proposed to examine the influencing factors in the willingness to adopt and practice telemedicine in Iraq. This study was prompted by a real need to examine the requirements, challenges, and gaps in adopting this new technology. A set of items of questionnaires was designed based on previous studies on the adoption of telemedicine in the Middle Eastern countries, such as Kuwait, Saudi Arabia, Jordan, and Syria. The pilot studies were conducted to examine each influencing factor in the hypotheses and to test the reliability of the questionnaire. The pilot study was required to verify the items. Cronbach's alpha test in the pilot study reveals that the factors have values more than 0.7, which are acceptable. The pilot study was conducted using questionnaires that were disseminated to physicians in Iraq. A research paper will follow this study to illustrate the results of the data analysis of the survey. Future research will then test the hypotheses and validate the framework. The results of the tests are expected to contribute to furthering the understanding and grasp of electronic information sharing in the healthcare sector. In addition, research of willingness to adopt telemedicine needs to be examined in rural area since it has different factors to measure.

## Figures and Tables

**Table 1:** Operationalization of the factors and items.

Factor	Items	References
Privacy	3 items	[17, 19]
Culture	3 items	[19–21]
Attitude toward telemedicine	3 items	[22, 23]
Benefit	5 items	[20, 21, 24]
Connectivity	3 items	[25, 26]
IT capability	3 items	[20, 21, 24, 27]
Compatibility	3 items	[5, 13, 28]
Data warehouse	3 items	[29–31]
Technical support	3 items	[21, 32]
Top management support	3 items	[5, 13, 28, 33]
Cost	4 items	[20, 24, 34]
Policy	3 items	[5, 13, 20, 21, 28]
Upper-level leadership	3 items	[5, 13, 35]

**Table 2:** Demographic characteristics.

	% of participants	
	Pilot study 1	Pilot study 1
Variable		
Male	24	80.0%
Female	6	20.0%
Less than 30	3	10.0%
From 30 to 40	21	70.0%
From 41 to 50	5	16.7%
More than 50	1	3.3%
Bachelor	21	70.0%
Master's	8	26.7%
Ph.D.	1	3.3%
Experience in years		
From 1 to 5	6	20.0%
From 6 to 10	14	46.7%
From 11 to 15	8	26.7%
More than 15	2	6.7%
Type of position		
Administrator	12	40.0%
Nonadministrator	18	60.0%

**Table 3:** Percentage and start/initiation time of consultation.

Variables	Number of participants	% of participants
*Percentage of consultation*		
Zero	5	16.7%
From 1% to 20%	19	63.3%
From 21% to 40%	5	16.7%
From 41% to 60%	1	3.3%
From 61% to 80%	0	0.0%
From 81% to 100%	0	0.0%
*Years of consultation*		
Zero	1	3.3%
Less than a year	9	30.0%
From 1 to 3 years	18	60.0%
From 4 to 6 years	2	6.7%
From 7 to 9 years	0	0.0%
10 years or more	0	0.0%

**Table 4:** Cronbach's alpha and number of items.

Factor	Cronbach's alpha	Number of items
Privacy	.756	3
Culture	.926	3
Attitude toward telemedicine	.755	3
Benefit	.729	5
Connectivity	.794	3
IT capability	.706	3
Compatibility	.763	3
Data warehouse	.749	3
Technical support	.787	3
Top management support	.706	3
Cost	.728	4
Policy	.815	3
Upper-level leadership	.818	3

**Table 5:** Part 3: individual characteristic.

	Strongly disagree	Disagree	Neutral	Agree	Strongly agree
Pri1: being concerned about patient privacy is important.					
Pri2: we have to keep the privacy of patients.					
Pri3: telemedicine is not private and confidential.					
Cul1: applying telemedicine will be negatively affected by culture and religion issues.					
Cul2: social issues (culture and religion) have a potential effect on applying telemedicine.					
Cul3: our culture and social norms refuse the use of telemedicine.					
ATT1: I trust technology to work.					
ATT2: I am happy using ICT/the Internet for patient care.					
ATT3: general comfort exists in using ICT/Internet to store, retrieve, and communicate patient information with other health institutions.					
Ben1: telemedicine enhances collaboration in the public sector.					
Ben2: telemedicine improves the healthcare service quality in Iraq.					
Ben3: telemedicine reduces cost in overall healthcare expenses.					
Ben4: telemedicine improves efficiency and resource utilization.					
Ben5: telemedicine improves access to healthcare services and care delivery, especially for people in rural and remote communities.					

**Table 6:** Part 4: technological characteristic.

	Strongly disagree	Disagree	Neutral	Agree	Strongly agree
Con1: the connectivity is important for healthcare delivery.					
Con2: physicians have to be connected to the Internet.					
Con3: physicians have to be connected to patients.					
ITc1: our healthcare requires information system applications.					
ITc2: our healthcare requires good ICT infrastructure.					
ITc3: telemedicine requires basic IT skills.					
Compat1: physician ICT is different from patient ICT.					
Compat2: telemedicine does not contrast with healthcare needs.					
Compat3: different telemedicine applications will not affect the efficiency.					
DWc1: we should keep patient health record perpetually.					
DWc2: there are needs of patient health record to have statistic.					
DWc3: we should store our patient's record in a common database to make them accessible.					

**Table 7:** Part 5: organization characteristic.

	Strongly disagree	Disagree	Neutral	Agree	Strongly agree
TS1: telemedicine requires good technical support.					
TS2: a specific focus on long-term sustainability is a common trait among successful programs.					
TS3: technical support provides measurement and feedback to physicians.					
TMS1: our top managers are interested in applying telemedicine.					
TMS2: our top managers have properly identified telemedicine needs.					
TMS3: our top managers have prioritized telemedicine needs.					
Cost 1: high cost of equipment might be the cause of not adopting telemedicine.					
Cost 2: telemedicine is less costly than traditional healthcare delivery.					
Cost 3: staff training is costly.					
Cost 4: software and hardware maintenance is costly.					

**Table 8:** Part 6: environmental characteristic.

	Strongly disagree	Disagree	Neutral	Agree	Strongly agree
Policy 1: the government should amend policies to support the telemedicine project.					
Policy 2: our hospitals require legislations and policies to apply the telemedicine project.					
Policy 3: legislations and policies build good relationship and trust among our staff.					
Upper 1: the Ministry of Health recommends the application of telemedicine.					
Upper 2: the Ministry of Health requests the application of telemedicine.					
Upper 3: the Ministry of Health provides the requirements to implement telemedicine.					

## Appendix

### Factors and Items

See Tables 5, 6, 7, and 8 and Figures 2 and 3.
